# Non-invasive assessment of ICP during infusion test using Transcranial Doppler Ultrasonography

**DOI:** 10.1186/2045-8118-12-S1-P6

**Published:** 2015-09-18

**Authors:** Danilo Cardim, Brenno Cabella, Joseph Donnelly, Chiara Robba, Marek Czosnyka, Matthew Garnett, John D Pickard, Zofia Helena Czosnyka

**Affiliations:** 1 Neurosurgery, University of Cambridge, UK; 2 Dept of Anesthesiology, University of Genoa, Italy

## Background

Transcranial Doppler (TCD) based methods have been used to estimate ICP noninvasively (nICP), however their relative accuracy varies between different types of intracranial hypertension: vasogenic, CSF circulatory or secondary to brain volumetric changes (oedema, contusion, hematoma, etc). This study aimed to compare four nICP methods in a prospective cohort of hydrocephalus patients whose CSF dynamics was investigated using infusion tests involving controllable test-rise of ICP.

## Methods

FV, ICP and non-invasive ABP were recorded in 53 patients diagnosed for hydrocephalus. nICP methods were based on: I) interaction between FV and ABP using black-box model (nICP_BB); II) diastolic FV (nICP_FVd); III) critical closing pressure (nICP_CrCP) and IV) TCD-derived pulsatility index (nICP_PI). Correlation between rise in ICP (∆ICP) and ∆nICP and averaged correlations for changes in time between ICP and nICP during infusion test were investigated.

## Results

All nICP formulas overestimated ICP at baseline (p<0.005): nICP_BB 10.76 (15.08-7.30); nICP_FVd 16.97 (22.56- 11.64); nICP_CrCP 18.34 (20.38-14.89); nICP_PI 16.57 (17.46-16.06). At plateau of ICP during infusion test, only nICP_BB and nICP_PI presented significant difference from ICP. From baseline to plateau, all nICPs estimators increased significantly (paired t-test, p<0.05). Correlations between ∆ICP and ∆nICP were better represented by ICPn_PI and ICPn_BB: 0.45 and 0.30 (p<0.05). nICP_FVd and nICP_CrCP presented non-significant correlations: -0.17 (p=0.21), 0.21 (p=0.13). For changes in ICP during individual infusion test ICPn_PI, ICPn_BB and ICPn_FVd presented similar correlations with ICP: 0.39±0.40, 0.39±0.43 and 0.35±0.41 respectively. ICPn_CrCP presented a weaker correlation (R=0.29±0.24). In those cases where changes of ICP related to vasogenic fluctuations (plateu wabes, B waves) overlapped rise related to CSF infusion, time- correlation between real and estimated ICP seemed to be remarkably better.

## Conclusions

Out of the 4 methods, nICP_PI was the one with best performance for predicting changes in ∆ICP during infusion test, followed by nICP_BB. nICP_FVd and nICP_CrCP showed unreliable correlations. Changes of ICP observed during the test were expressed by nICP values with only a moderate correlations. Vasogenic components of ICP seemed to be easier to estimate with TCD, than component related to increased CSF circulation.

